# Isolated Hypoglossal Nerve Palsy as an Early Symptom of a Granular Cell Tumor

**DOI:** 10.3390/ijerph19052690

**Published:** 2022-02-25

**Authors:** Juliana Lemound, Dimitrios Papadimas, Sabine Skodda, Andrea Tannapfel, Anriy Alekseyev, Martin Kunkel

**Affiliations:** 1 Department of Oral and Maxillofacial Surgery, Ruhr University Knappschaftskrankenhaus Bochum, 44892 Bochum, Germany; martin.kunkel@kk-bochum.de; 2 Department of Oral and Maxillofacial Surgery, Klinikum Saarbrücken, 66119 Saarbrücken, Germany; dimitriospapadimas1980@gmail.com; 3 Department of Neurology, Ruhr University Knappschaftskrankenhaus Bochum, 44892 Bochum, Germany; sabine.skodda@kk-bochum.de; 4 Department of Pathology, University of Bochum Medical Center, Ruhr University Klinikum Bergmannsheil Bochum, 44789 Bochum, Germany; andrea.tannapfel@pathologie-bochum.de; 5 Department for Radiology, Krankenhaus Wermelskirchen, 42929 Wermelskirchen, Germany; alekseyev@krankenhaus-wermelskirchen.de

**Keywords:** granular cell tumor, Abrikossoff, hypoglossal nerve, nerve palsy, tongue atrophy

## Abstract

Background: Hypoglossal nerve palsy (HNP) is rather common as a neurological disease. However, as an isolated nerve palsy it is an exceedingly rare phenomenon and points at local pathologies along the peripheral course of the nerve. In this communication we report a granular cell tumor (GCT) arising in the submandibular segment of the hypoglossal nerve. Case-Report: Spontaneous isolated HNP was recognized in a female patient. First line MR-imaging identified a clivus-chordoma. However, involvement of the hypoglossal nerve was highly unlikely according to MR-findings. Finally, ultrasonographic investigation revealed a small submandibular mass which, at histological examination, turned out to be a granular cell tumor arising within the hypoglossal nerve. Conclusions: This is the report of an extremely rare GCT originating within the 12th cranial nerve. The case illustrates that isolated motoric cranial nerve palsy may result from this rare tumor entity. This report also points out the diagnostic value of a simple ultrasonographic investigation to depict pathologic lesions of the submandibular space.

## 1. Introduction

Spontaneous isolated hypoglossal nerve palsy (HNP) is an exceedingly rare phenomenon and even when claimed to be isolated, detailed case reports most often describe either pain, meningeal signs and symptoms or cerebral and/or ocular ischemia [1,2,3,4,5,6]. This scarcity may be attributed to the close proximity of numerous important anatomical structures, which typically cause additional collateral symptoms rather than an isolated complaint. While quite common as part of neurological diseases involving the medulla oblongata, isolated hypoglossal palsy is rare and mostly indicative of local pathologies along the peripheral path of the nerve. Tumors or metastases of the skull base account for a major part of isolated HNP [7,8], as well as vascular pathologies, mostly internal carotid artery aneurysm or dissection [9,10,11]. A small fraction of cases are due to miscellaneous causes as dural arteriovenous fistulas [12], posterior fossa arachnoid cysts [13,14] and atlanto-occipital joint synovial cysts [3,15,16]. In addition, traumatic HNP has been described along with transoral intubation [17,18,19,20] and laryngeal mask application [21,22].

To the best of our knowledge, HNP caused by Abrikossoff’s tumor has been documented so far only by Xiang et al., 2016 [23]. Thus, we report a further rare finding of an isolated unilateral hypoglossal nerve paralysis caused by a granular cell tumor arising in the submandibular segment of the hypoglossal nerve.

## 2. Case Report

### 2.1. Clinical Findings and Imaging

**Figure 1:**
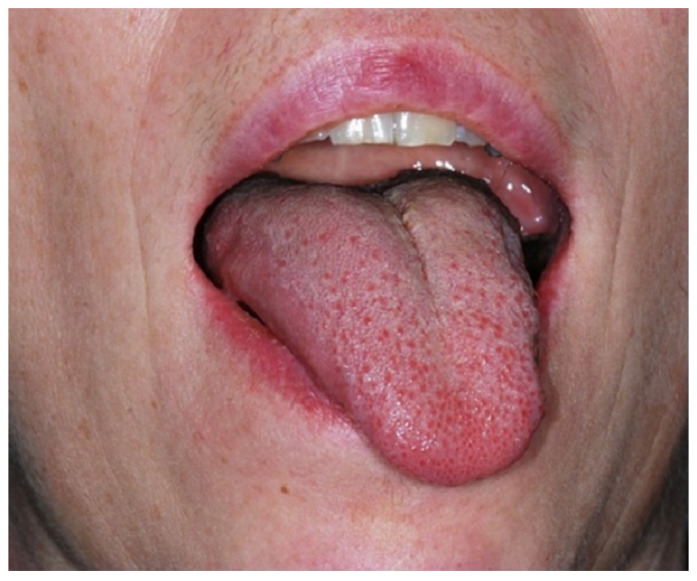
The initial clinical investigation shows a marked deviation of the tongue to the left as the typical feature of a left-sided hypoglossal nerve palsy.

The patient referred to our hospital was a 48-year-old Caucasian female who (as a former health care professional) noticed ongoing painless tongue dysfunction for about 6 months. On hospital admission, physical examination revealed deviation of the tongue to the left and ipsilateral atrophy as the typical feature of left-sided hypoglossal nerve palsy (Figure 1). There were no other clinical signs and symptoms, neither on neurological nor on maxillofacial examination. Past medical history included hypothyroidism and a fibroadenoma of the left breast. Maxillofacial trauma has not occurred and neither orotracheal intubation, previous regional surgery nor any interventional procedures have been performed prior to the onset of the symptoms.

**Figure 2:**
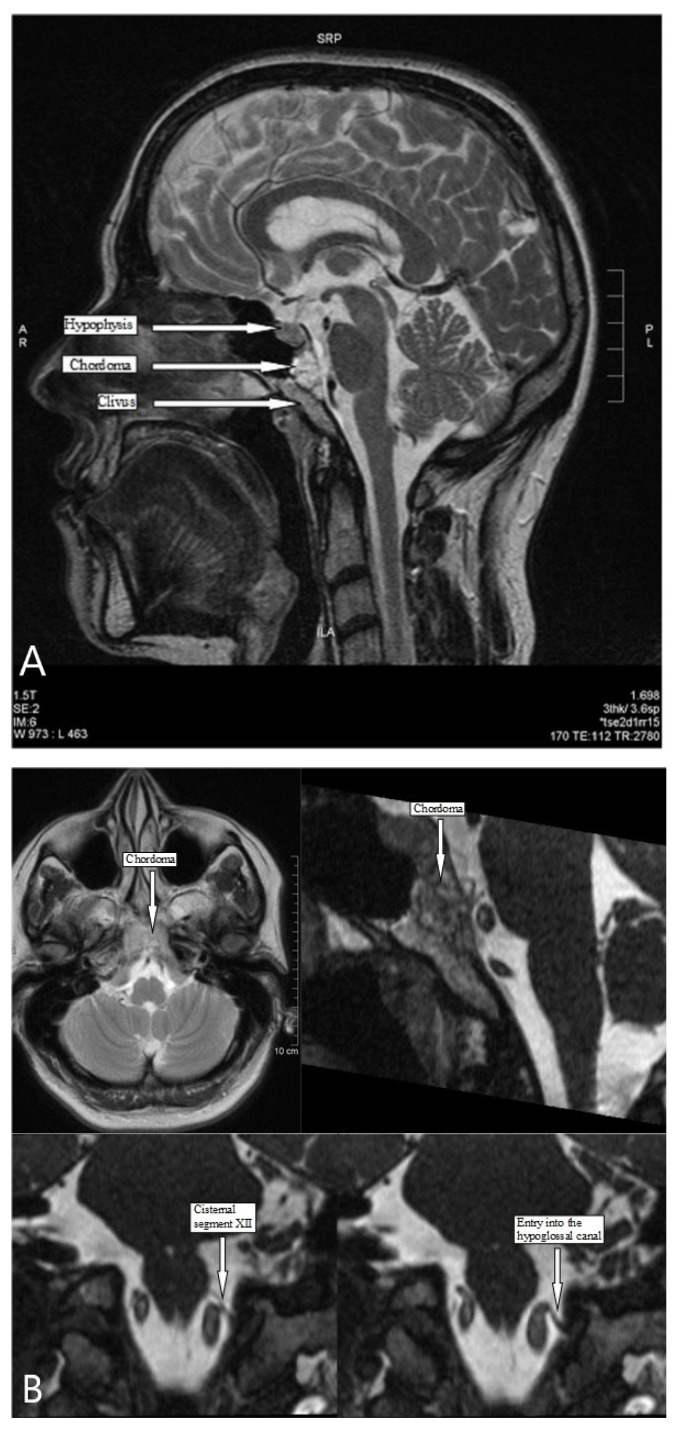
(**A**) MR-imaging, sagittal plane: Chordoma in the clivus area. (**B**) Cisternal segment and entry of the 12th cranial nerve into the hypoglossal canal.

Based on the initial neurological examination, an MRI of the neurocranium was performed. MRI imaging revealed an inhomogeneous, partly cystic, irregular, contrast-affinity lesion in the clivus with erosion of the cortical bone and incipient growth into the prepontine cistern, outside of brain parenchyma, suspected of a chordoma (Figure 2A,B). This was confirmed in cranial computed tomography in the bone window. No evidence of pathologies in the brainstem and in the cisternal course of the hypoglossal nerve up to the entrance into the hypoglossal canal were found. It was therefore not possible to determine any spatial relationship between the chordoma and the brainstem or the cisternal course of the hypoglossal nerve (Figure 2A,B). Thus, the chordoma could not be considered as the cause of palsy of the 12th cranial nerve.

**Figure 3:**
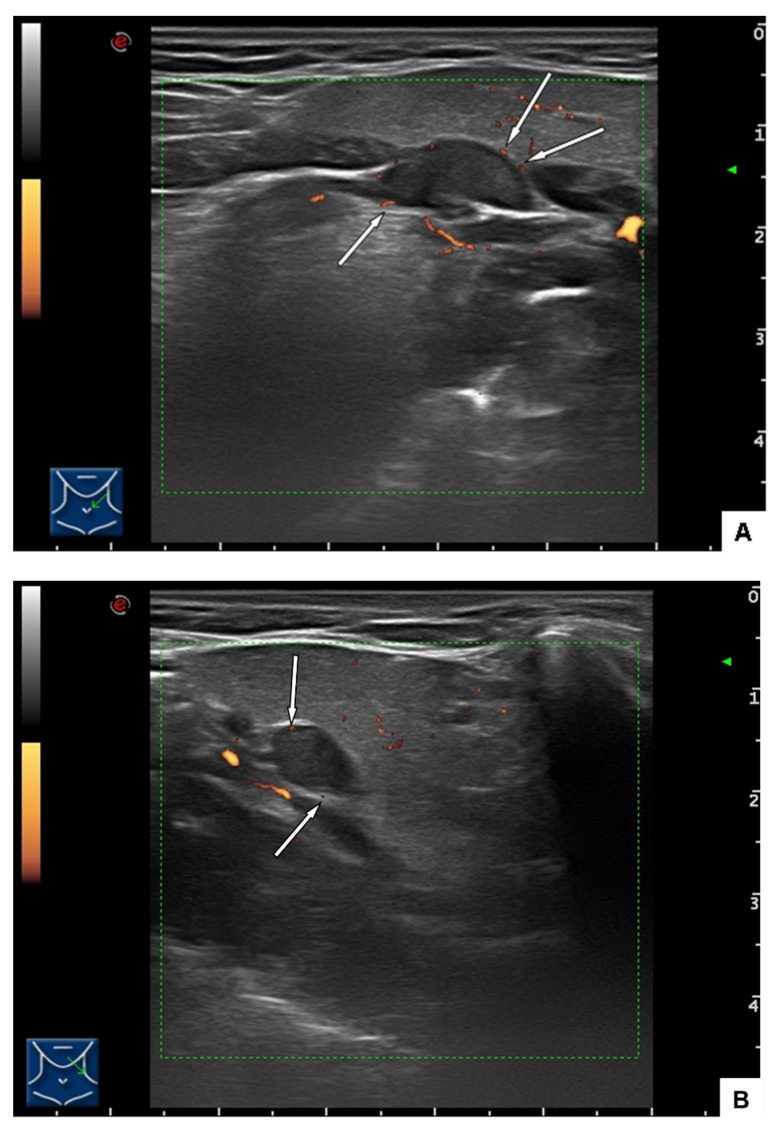
(**A**) Ultrasound findings: The rather homogeneous tumor is clearly demarcated from the surrounding tissues. Arrows indicate the position of vessels at the tumor surface. (**B**) Transverse view.

Consequently, in addition to the MRI of the neurocranium, an ultrasound examination was performed to clarify the morphological status of the hypoglossal nerve in its extracranial course. In the ultrasound scan (Figure 3A,B) was identified as a well-defined, rather homogeneous ovoid tumor measuring 14 × 7 × 6 mm along the path of the hypoglossal nerve, protruding into the submandibular space but clearly demarcated from the submandibular gland. Color flow mapping could not depict any vascular hilus structure but showed small vessels at the surface of the lesion. The nodal status of the neck was negative.

The ultrasound examination was explanatory for a peripheral hypoglossal nerve lesion, further examinations were not necessary.

### 2.2. Surgical Treatment and Outcome

**Figure 4:**
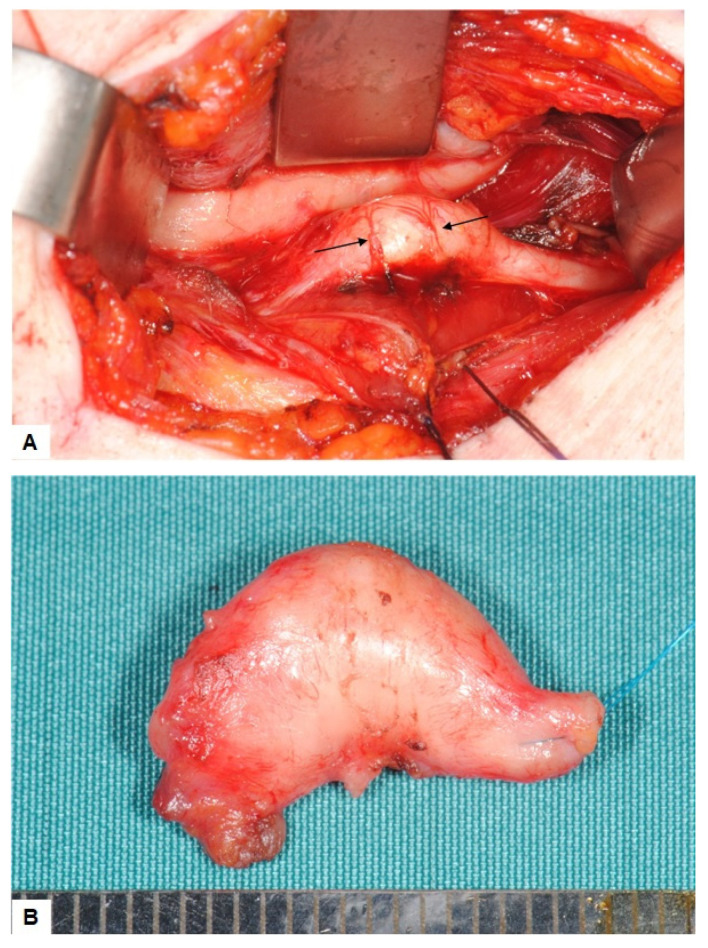
(**A**) Intraoperative findings: The tumor appears as a swelling of the hypoglossal nerve. As suggested by ultrasound findings, small vessels cross the surface. No adhesion to the surrounding tissues and especially no adhesion to the mylohyoid muscle is found. (**B**) Resection specimen: The tumor appears fully encapsulated by the perineurium When sectioned, there is no demarcation between the nerve and the tumor.

The mass was excised by a submandibular approach. Consistent to the preoperative ultrasonographic findings the lesion showed prominent superficial vessels (Figure 4A). The dissection finally exposed a yellowish tumor which appeared as a fusiforme swelling of the nerve, measuring about 15 mm. Since the tumor could not be separated from the nerve fascicles, complete surgical excision was performed (Figure 4B), followed by a simultaneous end to end anastomosis of the hypoglossal nerve. The postoperative clinical course was uneventful with partial recovery of the motoric function. There was no recurrence in the 20 months of follow up.

**Figure 5:**
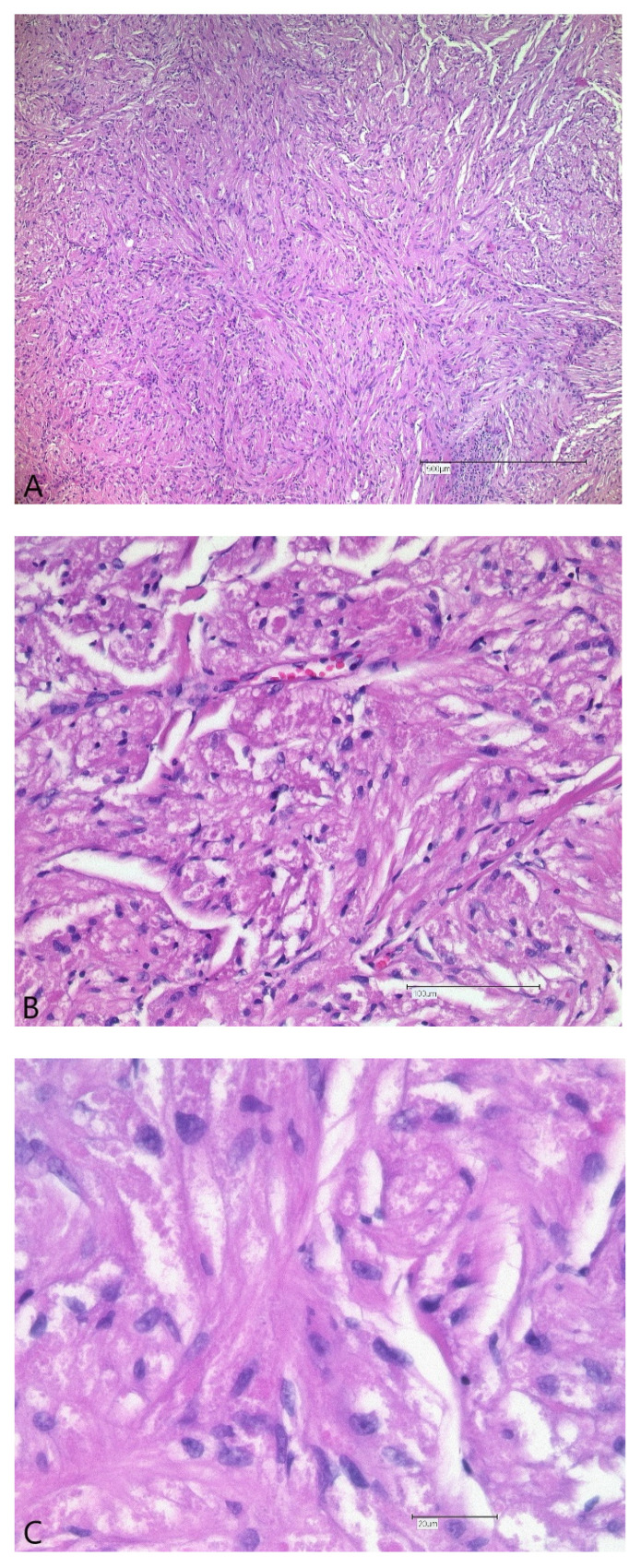

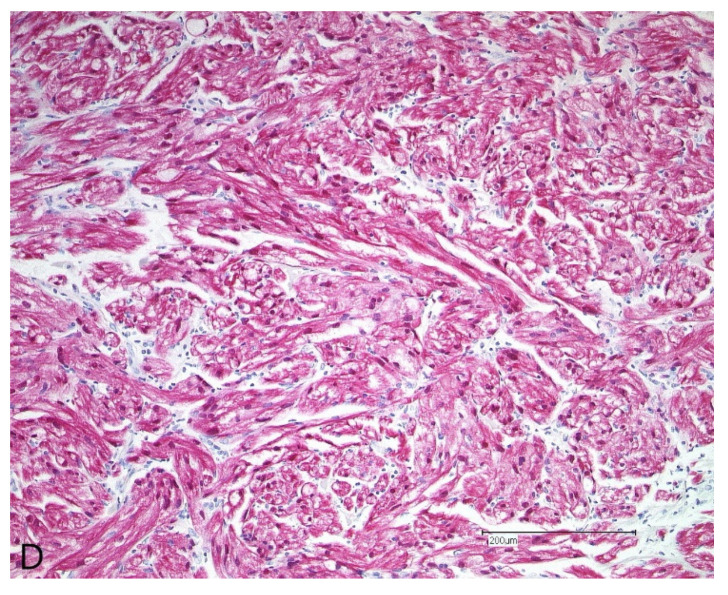
Histological findings: The granular appearance of the cytoplasm as to be seen at higher magnification is the typical morphological feature of GCT. H/E-staining, scale bars 500 μm (**A**), zoomed-in views 100 μm (**B**) and 20 μm (**C**). The tumor consists of aggregates of granular cells staining intensively positive for S100 ((**D**)**:** immunohistology for S-100). Granular cell infiltration and disruption of the nerval structures can be seen on both H/E-staining and after S-100 staining.

Histopathologic analysis showed a well-defined lesion consisting of clusters of polygonal cells with small nuclei and a characteristic granular eosinophilic cytoplasm. The HE morphology was suggestive of a granular cell tumor (GCT). Immunohistochemistry confirmed cytoplasmic granules to be strongly positive for S-100, typical for a GCT. The architecture of the hypoglossal nerve was completely disrupted, the nerve fascicles were split up. High-power magnification revealed diffuse infiltration of the nerve fascicles by S-100 positive granular cells (Figure 5A–D).

A few weeks later the pathologic mass in the clivus area was treated by a transnasal—transsphenoidal resection followed by subsequent proton irradiation. The histological analysis confirmed a clivus chordoma.

## 3. Discussion

Granular cell tumor is a rare neoplasm first described 1926 by the pathologist Dr. Alexei Abrikossoff [24]. The typical clinical appearance is a slowly growing, usually painless mass [25,26,27]. Although mostly benign, 1–2% of all GCT show a malignant phenotype arising either from a long-standing benign GCT or (mostly) de novo [28,29]. The GCT have a higher incidence during the third through the sixth decades of life and show a female preponderance in oral subsites [29,30,31,32,33]. About one half of the cases involve the head and neck, where it is predominantly found in the anterior two-thirds of the oral tongue [31,33,34,35]. Some cases were described in the gastrointestinal tract [36,37], in the upper respiratory tract [38], in the lower respiratory tract [39], in the breast [40] and extremely rare in peripheral nerves [41]. Peripheral occurrence has been reported for tibial [32], radial, ulnar [25,42], median, sciatic [28], sural [41] and digital nerves [43,44], all together either sensory or mixed type nerves. Regarding cranial nerves, involvement of trigeminal and recurrent laryngeal nerves has been found [32]. In contrast to this, 2016 a GCT of the hypoglossal nerve, presenting as an isolated unilateral hypoglossal nerve paralysis with tongue muscle atrophy has been reported by Xiang et al. [23]. In this case, the ca. 50 mm large tumor was identified in MRI as a non-encapsulated hypo signal mass posterior to the submandibular gland [23]. In our case, the 15 mm large tumor was diagnosticated by ultrasound scan, which was performed additional to MRI of the neurocranium.

It seems noteworthy, that GCT’s are generally considered to remain clinically silent [31] except for signs and symptoms caused by compression effects on the surrounding tissue or organs. In our case, however, the GCT caused highly specific symptoms such as a distinct motorial palsy and consecutive atrophy of the tongue resulting from the infiltration of the hypoglossal nerve. Similar clinical characteristics have only been reported for the rare cases, where GCT’s involve large peripheral nerves [25,45].

In case of isolated hypoglossal palsy, the recommended diagnostic workflow includes MRI of the neurocranium to identify pathologies in the brainstem and in the cisternal course of hypoglossal nerve. To investigate the peripheral extracranial course of the hypoglossal nerve ultrasound scan or MRI imaging of soft tissues of the neck and mouth floor are available.

The histogenesis of granular cell tumors has been a source of controversy for many decades. Due to the common intermuscular location and the cellular similarity between GCT and skeletal muscles, it was initially suggested that the tumor originates from embryonic myoblasts [30,31,46]. Pathological investigations including electron microscopic and immunohistochemical studies, however, failed to demonstrate a muscle cell origin and favored—due to the typical S-100 positivity—a neural derivation of the GCT, most likely from the Schwann cells [25,29,31,32,41,47,48].

Our findings are in line with the later hypothesis. The tumor was completely embedded in the perineurium without adherence or even contact to the neighboring muscles, thus, lacking signs of external infiltration. Despite this clear demarcation against surrounding tissue, virtually any fascicle of the nerve showed extensive infiltration by granular cells. Both the intraoperative and morphological features favor a neural derivation of the GCT.

## 4. Conclusions

In this report we present a rare case of a GCT originating within the hypoglossal nerve. The case illustrates that isolated motoric cranial nerve palsy may result from this rare tumor entity. The case also points out the diagnostic value of a simple ultrasound investigation to depict pathologic lesions of the submandibular space.

## Data Availability

The data are available upon request.
